# The Protease Inhibitor *CI2c* Gene Induced by Bird Cherry-Oat Aphid in Barley Inhibits Green Peach Aphid Fecundity in Transgenic Arabidopsis

**DOI:** 10.3390/ijms18061317

**Published:** 2017-06-20

**Authors:** Aleksandra Losvik, Lisa Beste, Sara Mehrabi, Lisbeth Jonsson

**Affiliations:** Department of Ecology, Environment and Plant Sciences, Stockholm University, 106 91 Stockholm, Sweden; aleksandra.losvik@su.se (A.L.); lisa@sunnerstascience.se (L.B.); sara.mehrabi@slu.se (S.M.)

**Keywords:** aphid resistance, serine protease inhibitor, *Myzus persicae*

## Abstract

Aphids are phloem feeders that cause large damage globally as pest insects. They induce a variety of responses in the host plant, but not much is known about which responses are promoting or inhibiting aphid performance. Here, we investigated whether one of the responses induced in barley by the cereal aphid, bird cherry-oat aphid (*Rhopalosiphum padi* L.) affects aphid performance in the model plant *Arabidopsis thaliana* L. A barley cDNA encoding the protease inhibitor *CI2c* was expressed in *A. thaliana* and aphid performance was studied using the generalist green peach aphid (*Myzus persicae* Sulzer). There were no consistent effects on aphid settling or preference or on parameters of life span and long-term fecundity. However, short-term tests with apterous adult aphids showed lower fecundity on three of the transgenic lines, as compared to on control plants. This effect was transient, observed on days 5 to 7, but not later. The results suggest that the protease inhibitor is taken up from the tissue during probing and weakly inhibits fecundity by an unknown mechanism. The study shows that a protease inhibitor induced in barley by an essentially monocot specialist aphid can inhibit a generalist aphid in transgenic Arabidopsis.

## 1. Introduction

Aphids are phloem feeding insects and serious crop pests. They are vectors of plant viruses and by themselves reduce plant growth at heavy infestations. Some species cause visible symptoms and even plant death [1]. The main control method to fight aphid pests is the usage of insecticides, which, however, have well-established negative effects, such as toxicity versus non-target organisms [2] and the development of insect resistance [3,4,5]. Because of such problems, regulations for insecticide usage are becoming increasingly restrictive [6,7]. The alternative approach of breeding for aphid resistance has been applied in several crops [8,9]. However, when based on single gene resistance, this approach faces the problem of the development of resistant aphid biotypes, such as in the case of the Russian wheat aphid [10]. In view of the need for durable resistance, more interest is now being directed to quantitative trait based resistance involving multiple loci, which is believed to be more durable [8,11,12].

The infestation by aphids induces a reprogramming of the gene expression in the host plant [8], something that has been studied in many plant/aphid species combinations (reviewed in, e.g., [9,13]). The interaction between *Arabidopsis thaliana* L. and the generalist green peach aphid (GPA) (*Myzus persicae* Sulzer) has emerged as a model system [13,14,15]. Genes induced in the Arabidopsis/GPA interaction and in other plant/aphid interactions have been further studied in transgenic and gene silencing approaches. The reducing effect on aphid population growth was confirmed in Arabidopsis for a lipase [16] and a gene involved in trehalose metabolism [17]. Likewise, for a α-dioxygenase upregulated in tomato by potato aphid, (*Macrosiphum euphorbiae* Thomas), the expression level was shown to correlate to numbers of potato aphids in tomatoes [18].

The present study is based on the similar assumption, i.e., that genes upregulated by aphids might confer aphid resistance. We have used a transgenic approach and the Arabidopsis/GPA interaction to investigate the effect of a protease inhibitor (PI), encoded by the barley (*Hordeum vulgare* L.) gene *CI2c*. There were several lines of evidence to suggest that *CI2c* might have a role in defense against aphids. First, *CI2c*, at the time named *BCI-7*, belongs to a small number of barley genes found to be induced in leaves by synthetic mimics of salicylic acid (SA) and by methyl jasmonate (MeJA) [19]. This is the background to the acronym *BCI*, meaning Barley Chemically Induced. Both SA- and JA-mediated responses have been reported in numerous plant–aphid interaction studies and there is suggestive evidence that they are important in efficient aphid defense [20].

A second reason to expect *CI2c* to be involved in aphid defense is its function. It is part of the *Mla*-locus, which confers resistance to powdery mildew and belongs to a family of six chymotrypsin inhibitor 2 (*CI2*) genes [21]. Plant protease inhibitors (PIs) are small proteins often induced upon pathogen or herbivore attack and have been much discussed as being important in plant defense against pathogens as well as insects [22,23,24,25,26]. It has since long been shown in several types of insects that PIs inhibit protein digestion in the gut and thereby their growth and reproduction [22]. With regard to aphids, some plant PIs have been shown to affect aphid performance. For example, some seed PIs of the cystatin, cysteine PI type have shown antibiosis effects against aphids in transformed plants [27,28,29], and the opposite effect has also been found [30]. There are also numerous studies with PIs added in artificial diets showing antibiosis effects against aphids, but usually at quite high PI concentrations [31,32,33,34]. However, most of the inhibitors tested have been seed or potato tuber PIs, which the aphids would not encounter on stems or leaves. Our study is the first where a sequence encoding a PI that is induced by aphids in leaf tissue is transformed to another plant species and evaluated for its effect against another aphid species. *CI2c* belongs to the potato inhibitor I family of serine protease inhibitors of the trypsin/chymotrypsin type [21]. The induction of chymotrypsin inhibitors in barley by aphids was shown as increased inhibitor activity long before transcript studies were carried out [35]. In our previous studies, *CI2c* was one of the genes found specifically induced by bird cherry-oat aphid (BCA) (*Rhopalosiphum padi* L.) in two moderately BCA resistant and not in two BCA susceptible barley genotypes [36]. In addition, the constitutive expression of *CI2c/BCI-7* was higher in a large selection of moderately BCA resistant barley genotypes in comparison to in susceptible genotypes [37]. This suggests that, in barley, the gene might be contributing to the multifactor BCA resistance, causing lower nymph growth [36]. Evidence for a function for serine proteases in GPA comes from a study where Arabidopsis was expressing ds-RNA of a GPA serine protease gene. Aphids feeding on these plants exhibited lower serine protease activity and a lower fecundity [38]. With this background, we hypothesized that the CI2c inhibitor might reduce the performance of GPA in Arabidopsis.

A PI may be acting in a plant/pathogen interaction by inhibiting either a plant protease or a protease from the pathogen and the effect may be either to the advantage or to the disadvantage of the pathogen [26]. A similar variety of scenarios can be expected in the plant/aphid interaction. Aphid proteases have been found in the aphid saliva [39] and the suggested function is in degrading phloem fiber proteins. This has been shown in vitro [40]. Proteases within the aphid body may be involved in digestive processes or in other functions related to protein turnover, reproduction or interaction with the bacterial symbionts. However, the PI may not necessarily act in a detrimental manner to the aphid. It is known that aphids manipulate plants to their own favor [41] and induced PIs might be favorable to the aphid by inhibiting plant proteases that are part of the plant defense machinery.

In order to find out whether CI2c would affect GPA on Arabidopsis, various bioassays were carried out on transformed plants expressing *CI2c* to examine host acceptance, fecundity and life span. The gene was expressed under control of either a constitutive promoter (*CaMV35S*) or the *rolC* promoter with a restricted expression, often cited as phloem-specific [42,43]. A change in host acceptance would indicate the recognition of the gene product during penetration of the aphid stylet in the tissue and probing. A short-term effect on fecundity would be an indication of an effect during probing or feeding establishment. Any long-term effect on fecundity or life span might indicate that the metabolism or other aspects of reproduction are affected. We used both bolting and non-bolting plants in several of the tests, since the interaction between GPA and Arabidopsis has been shown to differ depending on the growth stage of plants (rosette or flowering) [13,14]. In a parallel approach, we overexpressed *CI2c* in barley and studied the effect on BCA and GPA. It was found that this did not cause any effects on the performance of BCA, and that the overexpression caused GPA to prefer the transgenic line [44].

The main aim of the present study was to investigate whether the product of the *CI2c* gene induced by aphids in barley affects the generalist aphid GPA in another plant species. The major effect of overexpressing *CI2c* in Arabidopsis was a transient inhibition on GPA fecundity, indicating that the aphid encounters the CI2c protein during probing and that it inhibits metabolism or unknown aspects of reproduction.

## 2. Results

### 2.1. Confirmation of Transformation and Phenotypes of Arabidopsis Plants Expressing CI2c

**Figure 1:**
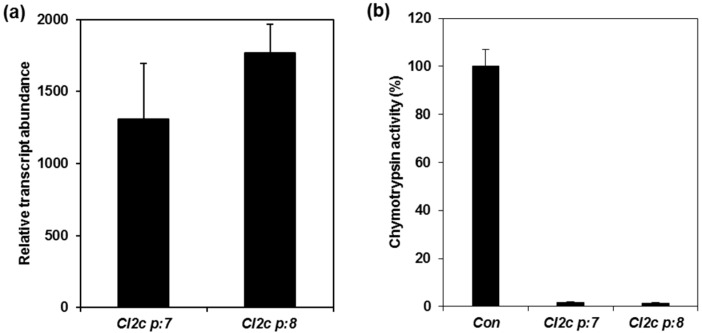

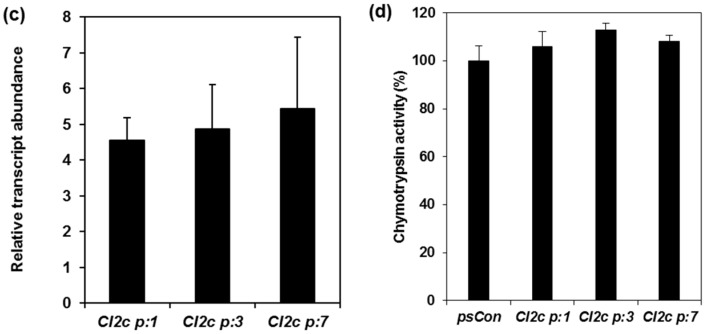
Transcript abundance and chymotrypsin inhibition in Arabidopsis expressing *CI2c*. Relative transcript abundance of the *CI2c* sequence in CI2c (**a**) and psCI2c (**c**) lines. The results represent the average (±SD) of four biological replicates. The transcript abundance was calculated relative to two reference genes: *Clathrin* and *TIP41*. The sequence was not found expressed in the corresponding azygous control lines. The inhibition of chymotrypsin activity in protein extracts from CI2c (**b**) and psCI2c (**d**) lines. Bars indicate average (± SD) of three technical replicates.

Two CI2c lines (CI2c p:7 and CI2c p:8) with constitutive expression of the transgene and three psCI2c lines with phloem-specific expression (psCI2c p:1, psCI2c p:3 and psCI2c p:7) were selected for experiments with aphids. Gene expression analysis revealed that the transcript abundance of the transgene was high in the selected lines with constitutive expression of *CI2c* (Figure 1a). The transcript abundance was, as expected, much lower in the psCI2c lines (Figure 1c). There was no expression of the transgene in any of the azygous lines selected as controls. Measurements of enzyme activity confirmed the presence of the gene product in the CI2c lines by strong inhibition of chymotrypsin activity as compared to control plants (Figure 1b). The transformation with phloem-specific expression did not result in detectable inhibition in the enzyme assays (Figure 1d), which can be explained by the limited tissue expression in these transformants.

The germination rate was lower in two of the psCI2c lines, but there were no significant differences in leaf area, percentage of flowering plants or time to flowering (Supplementary Figures S1 and S2, Table S1) or any other obvious phenotypic differences between transgenic lines and control plants (Supplementary Figure S3).

### 2.2. CI2c Expressed in Arabidopsis Has No or Minor Effect on GPA Settling

**Figure 2:**
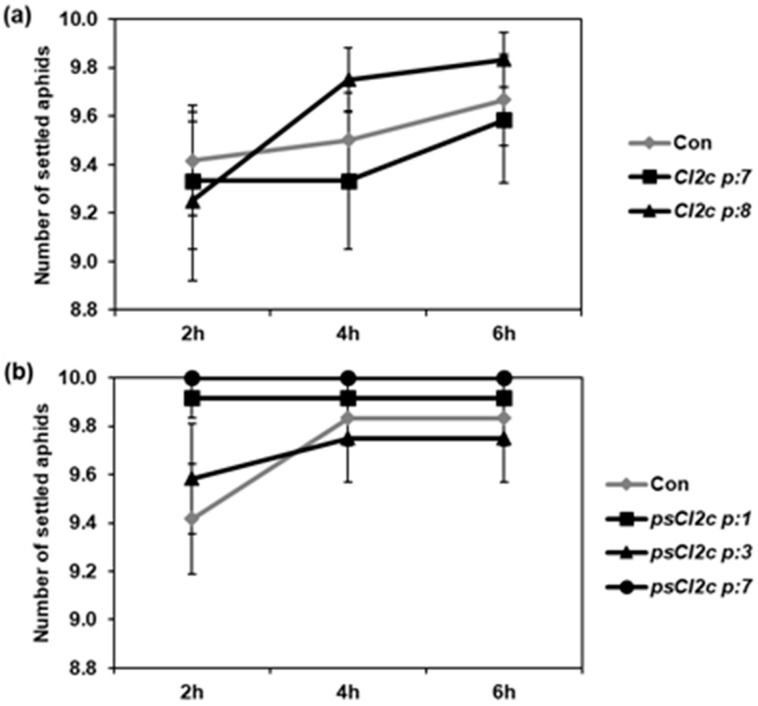
Proportion of GPAs settled in a no-choice test on (**a**) CI2c and (**b**) psCI2c lines and their respective controls. Ten adult apterous aphids were released in the center of the rosette of each Plant Results (±SE) (*n* = 12) represent data from two independent experiments. No significant differences were observed (*p* > 0.05 Kruskal–Wallis test).

**Figure 3:**
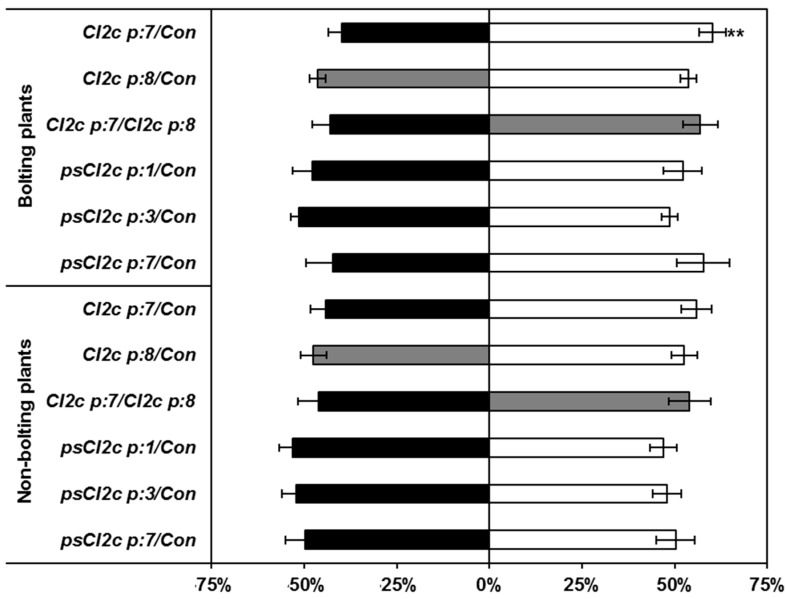
Percentage of GPAs settled in choice tests on CI2c and psCI2c lines or their controls. Twenty adult apterous aphids were released in the middle between two genotypes. The bars represent the percentage of aphids settled on each genotype. The data are from two independent experiments with number of replicates as given in the Section 4. Black or grey bars = transgenic lines as indicated; white bars = respective azygous controls. The asterisk indicates significant difference (** *p* ≤ 0.01 Wilcoxon signed rank test).

The no-choice test showed no difference between controls and transgenes in the proportion of aphids settled, at any of the time points 2, 4 and 6 h (Figure 2). In choice tests, a significant difference was observed in aphid settling in one combination only; control and CI2c p:7 plants (*p* ≤ 0.05, Wilcoxon signed rank test) for bolting plants (Figure 3). In the case of non-bolting plants, there was a consistent trend in aphid preference for control plants instead of CI2c p:7 and CI2c p:8 plants, but no significant difference. There was no difference in settling when the aphids had the choice between the two transgenic lines CI2c p:7 and CI2c p:8, or between the psCI2c transgenic lines and their respective controls (Figure 3). 

### 2.3. CI2c Expressed in Arabidopsis Has a Transient Short-Term Effect on GPA Fecundity

**Figure 4:**
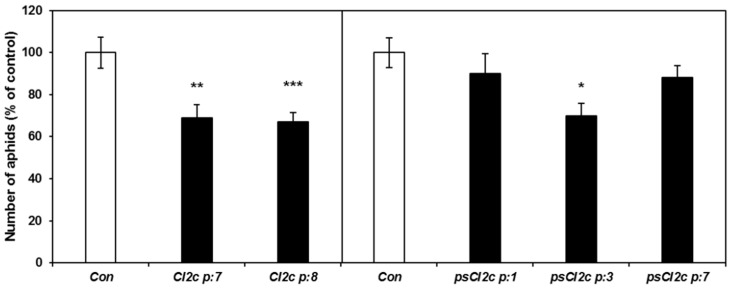
GPA fecundity on CI2c and psCI2c plants and on azygous controls. Five apterous adults were put on each plant and the total number of aphids on each plant counted after five days. The results (±SE) are from two independent experiments and normalized to the average aphid numbers on control plants for each experiment as 100 (these numbers were 48 and 26 for CI2c and 43 and 55 for psCI2c, respectively). Asterisks indicate significant differences between control and transgenic plants (* *p* ≤ 0.05; ** *p* ≤ 0.01; *** *p* ≤ 0.001, one-way ANOVA (*p* ≤ 0.05) followed by Tukey HSD as post hoc test).

**Figure 5:**
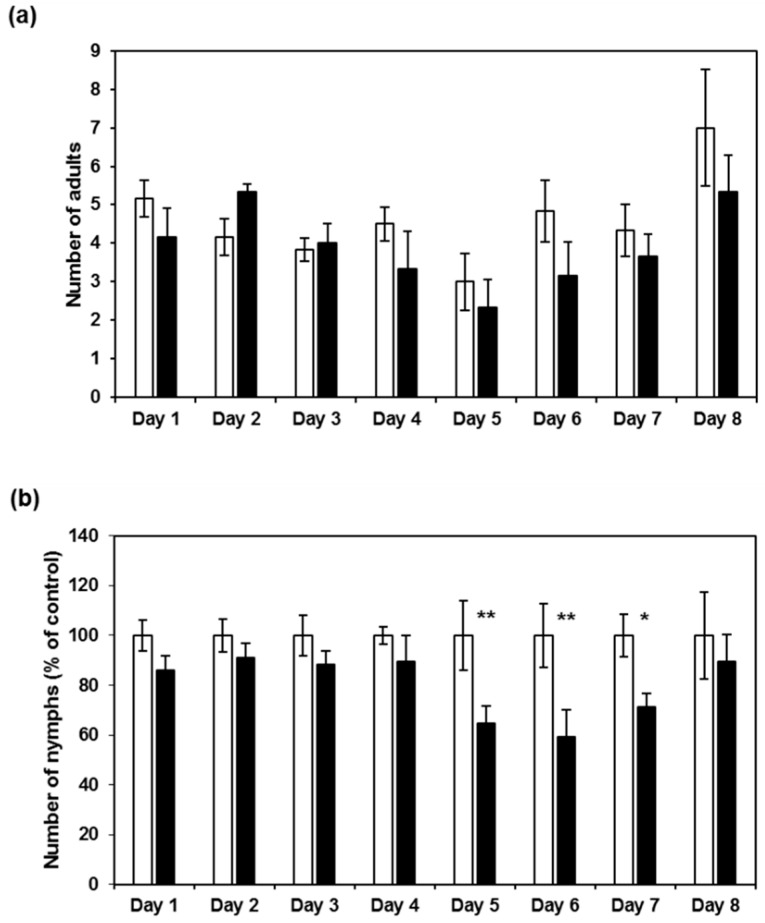

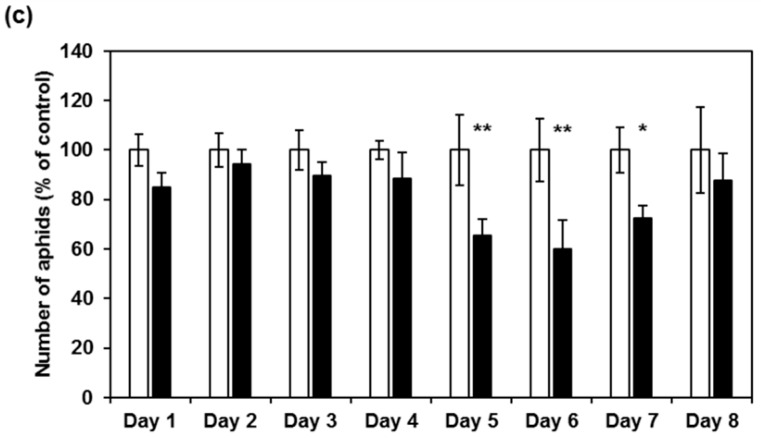
Time curve of GPA fecundity on CI2c p:8 and control plants. Five apterous adult aphids were released on each plant and the number of adult aphids and nymphs counted on a subset of the plants each day during eight consecutive days. (**a**) number of adults; (**b**) number of nymphs and (**c**) total number of aphids. White bars = control plants, black bars = CI2c p:8 plants. The results are the average (±SE). *n* = 6 for each day. For (**b**,**c**), the numbers were normalized to the average aphid numbers on control plants as 100. These numbers were 21, 41, 47, 68, 82, 34, 43, 47 for nymphs and 26, 45, 51, 72, 85, 39, 48, 54 for total aphids, respectively. Asterisks indicate significant differences between control and transgenic plant (* *p* ≤ 0.05; ** *p* ≤ 0.01, two-way ANOVA with line × day as factors followed by Tukey HSD as post hoc test).

To detect possible effects on aphid fecundity due to ingestion of the CI2c protein during penetration and probing, short-term fecundity tests were carried out. Adult aphids were added to bolting plants and the total numbers of aphids on each plant were counted after five days. The results showed lower numbers of aphids on the two *CI2c* transgenic lines (*p* ≤ 0.01 for CI2c p:7 and *p* ≤ 0.001 for CI2c p:8) and on psCI2c p:3 (*p* ≤ 0.05) as compared to on control plants (Figure 4). To further study the time scale of this effect, aphids were counted each day after settling during eight consecutive days in one of the transgene lines. The number of adults did not differ between the control and CI2c p:8 plants (two-way ANOVA: *F*_1,80_ = 3.38, *p* = 0.0697) (Figure 5a). It was the highest at day 8 when nymphs born at the beginning of the experiment reached adulthood and thus were counted as adults. The comparison of number of nymphs and the total number of aphids on control and CI2c p:8 plants showed that there were significant differences between the two lines (for nymphs: *F*_1,80_ = 18.1, *p* = 0.0001; for total aphid numbers: *F*_1,80_ = 16.76, *p* = 0.0001). There was no significant line × day interaction [nymphs: *F*_7,80_ = 0.92, *p* = 0.4927; total aphid numbers: *F*_7,80_ = 0.88, *p* = 0.5242]. Post hoc analysis revealed a significant decrease in the number of nymphs and total number of aphids on transgenic plants during the period of day 5 to 7 (*p* ≤ 0.01 for day 5 and 6 and *p* ≤ 0.05 for day 7) (Figure 5b,c).

### 2.4. CI2c Has No Long-Term Effect on GPA Fecundity

In order to find out if CI2c would have an effect on aphids when they have settled and are feeding from the phloem, long term experiments were carried out. In life span experiments, both the life span and the fecundity during the total life span are recorded starting with the birth of each nymph. The results showed no differences between transgenic lines and the control plants in the number of days for aphids to the start of reproduction, the length of the reproductive life, the life span, the number of nymphs produced per individual, the number of nymphs per day or the intrinsic rate of population increase *r*_m_ (Table 1).

**Figure 6:**
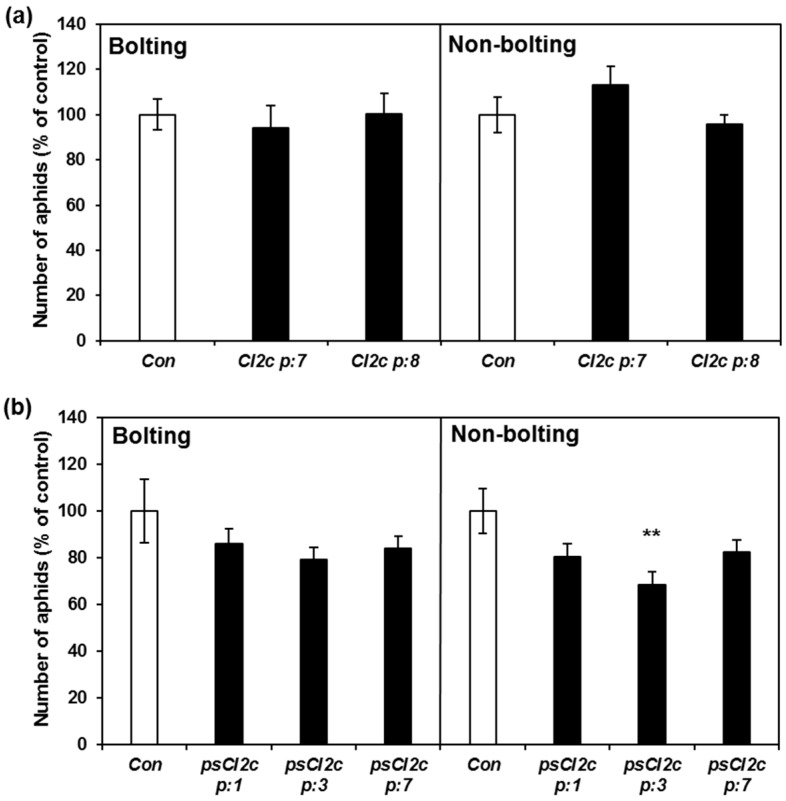
GPA fecundity on (**a**) CI2c and (**b**) psCI2c lines and their respective controls. Five nymphs were put on each plant and the total numbers of aphids on each plant were counted after 14 days. The data are from two independent experiments. Results (± SE) are normalized to the average aphid numbers on the control plants for each experiment as 100. The average numbers on control plants were: CI2c, bolting: 120 and 135; non-bolting 53 and 91; psCI2c, bolting 176 and 159; non-bolting 77 and 92. Asterisks indicate significant differences between control and transgenic lines at ** *p* ≤ 0.01 (one-way ANOVA followed by Tukey HSD).

Another type of fecundity test starting with nymphs was carried out during 14 days on both bolting and non-bolting plants. Considering that the days to the first reproduction is eight to nine days (Table 1), only one generation of nymphs are born within this time. The results showed lower aphid reproduction on the non-bolting line psCI2c p:3 in comparison with the controls (*p* ≤ 0.01), but not on any of the other transgenic lines or growth conditions (Figure 6).

## 3. Discussion

*A. thaliana* was transformed with *CI2c* under control of the constitutive *CaMV35S* promoter and in addition, the phloem-specific promoter *rolC*. The *rolC* promoter has been shown to direct the expression to phloem parenchyma and companion cells in tobacco [42] and in stems of *A*. *thaliana* to primary phloem and a random selection of cortical cells [45]. We found much lower transcript abundance of *CI2c* using the *rolC* promoter than the constitutive *CaMV35S* promoter (Figure 1), which is in agreement with the expected tissue-specific expression of the transgene.

We considered that differences in plant size or development between transgenes and control plants might affect the nutritional value and thus cause secondary effects on aphid performance. We find this very unlikely since there were no phenotypic differences between the transgenic and control plants at the age of the aphid tests. The only difference found was reduced seed germination in two of the psCI2c lines, possibly caused by the inhibition of storage protein degradation needed for germination in these seeds. The possibility of “seed history” effects, i.e., that induced defenses in the previous generation may have been carried through to the plants to be tested [46] was avoided by using azygous controls from the same line and from the same generation as the transgenic plants. Thus, the plants used for comparisons had experienced the same growth conditions in several generations.

A first consideration when analyzing the results is whether the PI stays within the cytosol or is secreted to the apoplast. In the first case, it may be delivered symplastically from the companion cells to the sieve cell elements, since this is the presumed trafficking pathway for proteins found in the sieve cell elements [47]. In this case, it would be present in the phloem sap from which the aphid is feeding. If, however, the PI has an extracellular location, it is unlikely to enter into the phloem sap because it would have to pass the plasma membrane. Many of the plant protease inhibitors are secreted to the extracellular space [24,25,26]. The CI2c sequence does not contain a signal peptide, but it has been suggested that, for certain small proteins, a signal peptide is not necessary for entry into the secretory pathway [48]. As an example, the closely related CI2, present in barley endosperm, did not contain any signal peptide [49] and yet was directed from the cytosol into protein bodies [50]. Based on the above, we presume that CI2c is secreted out of the cell.

There are several stages during aphid infestation where a PI might affect aphid performance. One possibility is that the PI has its main effect during aphid probing. The major factors determining aphid preference are encountered after stylet penetration of the peripheral host plant tissues, but before phloem ingestion [51,52]. The stylet follows an apoplastic pathway to the phloem and on its way briefly punctures many cells. The aphid quickly withdraws the stylet upon puncturing cells, but a mixture of saliva and cytoplasm is ingested. Will and coworkers [53] showed that the aphid saliva composition is different depending on the milieu of the stylet tip and concluded that aphids must permanently take up plant fluids, both from the apoplast and the cytosol. The results from our study do not give support to a strong effect of CI2c on host acceptance, since there was no delay in aphid settling on the transgenic lines in the no-choice tests and evidence for non-preference in the choice test was found with only one of the transgenic lines (Figure 3).

A second possibility is that ingestion of the CI2c during probing has a negative impact on the metabolism or reproduction and, as a consequence, the fecundity. Our results support this idea, since we found transient lower fecundity in three transgenic lines as compared to control lines. The effect was found with adults in three transgenic lines but only with one line using nymphs, suggesting that reproductive mechanisms are targeted. In the life span test, the mother has established feeding in the phloem. The finding that there were no differences in fecundity or life span between plants expressing *CI2c* and control plants in this test indicates that the CI2c protein is not ingested from the phloem, and supports the idea that it is an extracellular protein.

The *CaMV35S* promoter directs expression to both phloem and other tissue in dicots [54,55] and the expectations from transformation with the *rolC* promoter was to distinguish effects mainly restricted to phloem tissue to those in other parts of the leaf. As an example, in an earlier report, tobacco and chickpea were transformed with a gene encoding a leaf agglutinin protein using either *CaMV35S* or *rolC*-directed expression and the effects evaluated with GPA nymphs (on tobacco) or *Aphids craccivora* nymphs (on chickpea) [56]. The results showed somewhat higher nymph mortality on the *rolC*-transformants than those transformed with the *CaMV35S*-promoter [56]. Contrary to the CI2c protein, the protein in that study caused strong toxicity, since less than 50% of the nymphs survived, the 72 h test [56]. Our present results do not show any strong effects related to the use of promoter. It may be noted that, despite much lower total transcript abundance and activity of CI2c than in the lines with constitutive expression, the psCI2c p:3 line supported lower fecundity both in the short term (five days) and the 14 days tests starting with nymphs.

The transient effects on aphid fecundity that we found might be caused by targets in the aphid saliva or within the aphid. One target might be the gel saliva that forms a sheath in the apoplast during stylet penetration. It was shown with pea aphid (*Acyrthosiphon pisum* Harris) that, when the sheath protein is downregulated and no sheath can be formed, the long-term reproduction is suppressed [57]. Since this effect was not seen in our experiments, it seems unlikely that the gel saliva was affected. Alternatively, proteases in the watery saliva injected into the sieve cell elements might be inhibited. Recent studies showed that saliva from pea aphid and potato aphid digested the *Cucurbita maxima* Duchesne PP1 sieve-tube protein, and it was suggested that PIs in the phloem sap might protect the phloem proteins by inhibiting the saliva proteases [40]. In this scenario, GPAs might successfully establish feeding, but the inhibition of proteases by CI2c might delay reproduction. The argument against this idea is that we would then expect inhibitory effects during the life span tests where aphids have established feeding, and no such effects were found. We therefore suggest that the CI2c protein is ingested during probing and that the targets are within the aphids.

This idea would be in accordance with recent studies where lower serine protease activities in the GPA gut were correlated to lower fecundity [38]. It is also possible that the PI does not act on protein digestion in the gut, but, for example, on functions related to aphid reproduction or the interaction with the bacterial endosymbionts. This was suggested based on the finding that in GPA reared on oilseed rape expressing a gene for the cysteine PI oryzacystatin (*OC-I*), the inhibitor was found in the oenocytes and bacteriocytes of the aphid [27]. More recently, it was demonstrated that a cathepsin-L-like protease purified from the midgut of bean bug (*Riptortus pedestris* Fabricius) had antibacterial activity against gut symbiotic bacteria [58].

In barley overexpressing *CI2c*, GPA preferred the transgenic plants compared to controls, possibly due to protection against a serine protease found to be upregulated by GPA in barley [44]. The transcripts induced by GPA in Arabidopsis do not seem to include any serine proteases [59]. This may explain the absence of any preference of GPA for Arabidopsis expressing the *CI2c* gene.

## 4. Materials and Methods

### 4.1. Plant Cultivation

Seeds of *A. thaliana* were surface sterilized in a three-step procedure. Seeds were first kept for 1 h in a refrigerator in water with a few drops of Triton X-100 (Sigma-Aldrich Chemie GmbH, Steinheim, Germany), then washed during 20 min in 1:3 diluted NaClO-based commercial bleach (Klorrent, Nilfisk Advance, Brøndby, Denmark) and finally washed for 30 s in 70% ethanol. After each step, the seeds were rinsed with sterile water to remove the sterilizing agent. Plants were grown in growth chambers at 150 μmol photons m^−2^·s^−1^ and 22 °C. To obtain bolting plants, seeds were sown on plates with half-strength Murashige and Skoog (MS) medium and 0.25% (*w*/*v*) Gelrite (both from Duchefa Biochemie, Haarlem, the Netherlands) and transferred to a growth chamber with long day (LD) conditions (L16:D8). After 14 days, plants were transferred to 6 cm × 6 cm pots with soil (Blomjord, Hammenhög, Sweden) and grown under the same conditions for 10 more days. To obtain non-bolting plants, surface-sterilized seeds were sown directly into pots (6 cm × 6 cm) containing soil and placed in a growth chamber with short day (SD) conditions (L8:D16). After four weeks, the seedlings were replanted to 7 cm × 7 cm pots.

### 4.2. Aphid Rearing

Individuals of green peach aphid, *Myzus persicae* Sulzer (Aphididae, Hemiptera) were collected in the field near Uppsala, Sweden. Aphids were reared on kohlrabi (*Brassica oleracea* L. cv. Delikatess weisser) in a growth chamber with SD conditions as described above.

### 4.3. Plasmid Constructs, Plant Transformation and Selection

RNA was extracted from the doubled haploid barley breeding line 5172-28:4 [36] using the NucleoSpin^®^ RNAII kit (Macherey-Nagel, Düren, Germany) following the manufacturer’s instructions. Three µg of RNA was used for synthesis of first strand cDNA using the Transcriptor High Fidelity cDNA Synthesis Kit (Roche Diagnostics GmbH, Mannheim, Germany) according to the manufacturer’s instructions. The ORF of the barley PI (*CI2c*) was amplified from the cDNA using the primers 5′-CACCATGAGCTGCGCCGCC-3′ and 5′-TTGCAAAGCTAGCTAGCCAATGTGG-3′. Phusion high-fidelity DNA polymerase (Thermo Scientific, Vilnius, Lithuania) was used for the PCR reaction at 98 °C for 30 s, 35 cycles at 98 °C for 10 s and 72 °C for 30 s followed by 72 °C for 7 min. The PCR product was cloned into the Gateway^®^ pENTR/D-TOPO cloning vector (Invitrogen, Life technologies Corporation, Carlsbad, CA, USA). For constitutive expression with the cauliflower mosaic virus (*CaMV*) 35S promoter, the PCR product was introduced by LR clonase reaction (Invitrogen) in the destination vector pK7WG2 [60]. For phloem-specific expression, the *Agrobacterium* *rhizogenes rolC*-promoter was amplified using primers 5′-AGCGAAAGGATGTCAAAAAAGGATGC-3′ and 5′-ATGGTAACAAAGTAGGAAACAGGTTGC-3′ from plasmid pPCV7002-rolBC and cloned into the Gateway^®^ pENTR5′TOPO vector (Invitrogen). The PCR products (promoter in pENTR5′TOPO and cDNA in pENTR/D-TOPO) were introduced in the destination vector pK7m24GW.3 [61] using the LR Clonase II plus enzyme mix (Invitrogen). The binary vectors were transformed into *Agrobacterium tumefaciens* strain C58pGV2260 and used to transform plants of *A. thaliana* ecotype Colombia by the floral dip method [62] at the Uppsala Transgenic Arabidopsis Facility, Sweden. Transformants were selected on medium containing kanamycin (50 µg·mL^−1^, Sigma-Aldrich), self-fertilized, and analyses were performed on T3 or T4 lines homozygous for a single-gene insertion. Controls were azygous lines selected at T3.

### 4.4. Analyses of Transcript Abundance

For RT-qPCR studies, plant material was frozen in liquid nitrogen and stored at −80 °C until used for RNA isolation. Total RNA was extracted from three leaves of seven to eight weeks old plants. RNA extraction, reverse transcription, qPCR conditions and calculations of relative transcript abundances were performed as described in [37]. *Clathrin* and *TIP41* were used as reference genes [63]. A standard curve was made for each primer pair to calculate the efficiency of the primer in the reaction. The primer sequences are presented in Supplementary Table S2.

### 4.5. Enzymatic Assay of Protease Inhibitor Activity

Six weeks old non-bolting Arabidopsis plants were frozen in liquid nitrogen and stored at −80 °C. Whole shoot tissue was ground in a cold mortar on ice, with 0.1 M potassium phosphate buffer (3 mL buffer g^−1^ tissue) containing 5% polyvinylpolypyrrolidone (Sigma-Aldrich) and 0.1 M β-mercaptoethanol, pH 7.1. The samples were centrifuged at 12,000× *g* for ten minutes at 4 °C. The supernatants were cleaned from low molecular weight compounds on PD-10 columns (GE Healthcare, Little Chalfont, UK), eluted with 0.1 M phosphate buffer, pH 7.1. The protein concentration was determined using Bradford reagent (Sigma-Aldrich) with bovine serum albumin (Sigma-Aldrich) as standard. Protease activity in the extracts was measured with *N*-succinyl-Ala-Ala-Pro-Phe-7-amido-4-methylcoumarine (Sigma-Aldrich) as the fluorogenic substrate. The substrate was solubilized in dimethyl sulfoxide (Sigma-Aldrich) and then diluted in 0.1 M Tris-HCl (pH 8.0). The chymotrypsin inhibition assay in a final volume of 0.2 mL contained 60 µL of plant protein extract (final protein concentration of 24 µg·mL^−1^), 20 µL of chymotrypsin (Sigma-Aldrich; final concentration of 60 ng·mL^−1^) and 20 µL of substrate (final concentration of 40 µM). The plant extract was first incubated for 10 min at 30 °C with chymotrypsin in 0.1M Tris-HCl buffer pH 8.0. Then, the fluorogenic substrate was added and the fluorescence signal was measured in a Hidex Sense (Hidex, Turku, Finland) microplate reader for 30 min at 30 °C using a 390 nm excitation filter and an emission filter of 460 nm. Each reaction was prepared in triplicate. Enzyme activities were calculated from 10 min of initial linear velocity rates.

### 4.6. Aphid Settling

No-choice tests of aphid settling were carried out on 24 days old bolting plants in the light. Ten adult apterous aphids were released in the center of the rosette on each plant and the total numbers of aphids settled (i.e., not moving) on each plant were counted at 2, 4 and 6 h after their release. The experiments were carried out in two independent experiments (*n* = 6 in each). In host choice tests, the different genotypes (24 days old bolting plants or 6–7 weeks old non-bolting plants) were planted in opposite corners of the same square pot (7 cm × 7 cm). Twenty apterous adult aphids were carefully released in the middle of a dry filter paper (3 cm diameter) that was placed between the plants (Supplementary Figure S4). The plants were kept in a growth chamber with SD conditions as described above and the aphid location was assessed after 24 h. The choice tests between transgenes and controls were carried out two times on bolting and non-bolting *CI2c* (*n* = 15 or 16) and on non-bolting *psCI2c* plants (*n* = 12) and once on bolting *psCI2c* plants (*n* = 11). Tests with the choice between two *CI2c* transgenic lines were carried out once for bolting plants (*n* = 5) and twice for non-bolting plants (*n* = 17).

### 4.7. Aphid Fecundity and Life Span

To determine short-term effects on fecundity, five apterous adult aphids were released on each transgenic or control plant (bolting plants, 24 days old), and, after five days, the total numbers of aphids on each plant were counted. Plants were in open trays. The experiment was carried out in two independent experiments (*n* = 6 to 8 in each). To follow the fecundity day by day during eight days, five synchronous apterous adult aphids (9–10 days old) were released on *CI2c p:8* and control plants. Adults and nymphs were counted each day during eight days, each day from a new set of plants. This experiment was carried out once (*n* = 6 for each day). In the test to determine fecundity during 14 days, five one-day old nymphs were transferred to each transgenic or control plant (bolting, 24 days old or non-bolting, seven to eight weeks old). Plants were in a growth chamber with SD conditions as described above under transparent cages made of plastic and fabric (Supplementary Figure S4). The experiment was carried out in two independent experiments with 12 to 14 replicates.

To determine fecundity during the life span, one apterous adult aphid was placed on one of the young leaves (non-bolting plants, seven to eight weeks old) (six to eight replicates, each on a different plant). All selected leaves were similar in size and age. The leaf was enclosed in a transparent plastic container (5 cm high, 7 cm in diameter) with a small hole for the leaf (Supplementary Figure S4). Aeration was created by small holes in the plastic. The space around the leaf hole was closed with a piece of cotton wool to prevent aphid escape (Supplementary Figure S4). When the first offspring was produced, the aphid was removed together with all but one newborn nymph. For each single nymph, its reproduction and life span was monitored. All nymphs produced were counted and removed daily. During the period of the experiment, plants were kept in a growth chamber under SD conditions as described above. At the start of these experiments, plants were not flowering, but towards the end, flowers were developing. The intrinsic rate of population increase (*r*_m_) was calculated according to [64] as 0.738 (log *M*_d_)/*d*, where *M*_d_ is the number of progeny produced by an aphid in a period equal to the pre-reproductive time and *d* is the pre-reproductive time in days.

### 4.8. Statistical and Sequence Analyses

Normal distribution of data was analyzed using the Shapiro–Wilk normality test and was confirmed for fecundity tests, but not for aphid settling and life span test. Analyses of differences in aphid settling were carried out using a Kruskal–Wallis test (no-choice test) or by Wilcoxon signed rank test (choice tests) at *p* ≤ 0.05. Differences in aphid fecundity (five days test and 14 days test) were analyzed using one-way ANOVA (*p* ≤ 0.05) followed by Tukey HSD as a post hoc test, at *p* ≤ 0.05. Differences in aphid fecundity measured over a period of eight days were analyzed using two-way ANOVA with “Line” and “Day” as fixed factors followed by Tukey HSD as post hoc test at *p* ≤ 0.05. Results from life span experiments were analyzed using a Kruskal–Wallis test. All statistical analyses were performed with StatPlus Pro v5 for Windows from AnalystSoft Inc (Walnut, CA, USA). The presence of a signal peptide in the CI2c amino acid sequence was analysed using SignalP 4.1 (http://www.cbs.dtu.dk/services/SignalP/) [65].

## 5. Conclusions

The *CI2c* gene, which is encoding a chymotrypsin inhibitor in barley, reduced certain aspects of GPA performance when its cDNA was expressed in Arabidopsis. The most consistent effect was a transient inhibition on the fecundity of adult aphids. We suggest that this is caused by ingestion of the CI2c protein during probing, causing inhibitory effects in the aphid or its symbionts.

## Figures and Tables

**Table 1:** Life span and reproduction of green peach aphid (GPA) on CI2c and psCI2c p:3 lines. The results represent mean values (±SE). *r*_m_ = intrinsic rate of population increase. There were no significant differences between control and transgenic plants for any of the parameters (Kruskal–Wallis test, *p* > 0.05).

	Control (*n* = 6)	CI2c p:7 (*n* = 6)	CI2c p:8 (*n* = 6)	Control (*n* = 9)	psCI2c p:3 (*n* = 9)
Days to reproduction	8.7 ± 0.2	8.4 ± 0.3	8.7 ± 0.2	8.7 ± 0.2	9.1 ± 0.3
Reproductive life (days)	23.6 ± 2.0	22.7 ± 1.8	24.2 ± 1.4	24.4 ±1.7	25.3 ±1.5
Life span (days)	42.0 ± 4.0	41.1 ± 3.1	42.8 ± 2.7	49.1 ±3.4	46.6 ± 3.9
Nymphs/individual	63.9 ± 4.4	59.3 ± 2.7	59.7 ± 3.8	49.0 ± 6.1	38.9 ± 2.5
Nymphs/day	2.8 ± 0.1	2.8 ± 0.3	2.6 ± 0.3	2.0 ± 0.3	1.6 ± 0.1
*r*_m_	0.31 ± 0.004	0.31 ± 0.008	0.30 ± 0.010	0.26 ± 0.012	0.23 ± 0.014

## Supplementary Materials

Supplementary materials can be found at www.mdpi.com/1422-0067/18/6/1317/s1.

## Abbreviations

BCA	Bird cherry-oat aphid
CI2c	Barley chymotrypsin inhibitor 2c
GPA	Green peach aphid
PI	Proteinase inhibitor
