# Supplementary material for: The Protease Inhibitor CI2c Gene Induced by Bird Cherry-Oat Aphid in Barley Inhibits Green Peach Aphid Fecundity in Transgenic Arabidopsis
Source: Int J Mol Sci. 2017 Jun 20;18(6):1317. doi: 10.3390/ijms18061317 (PMC5486138; doi:10.3390/ijms18061317)
Supplement: Supplementary file 1 [file ijms-18-01317-s001.docx]

Supplementary Materials: The Protease Inhibitor *CI2c* Gene Induced by Bird Cherry-Oat Aphid in Barley Inhibits Green Peach Aphid Fecundity in Transgenic Arabidopsis

Aleksandra Losvik ^†^, Lisa Beste ^†^_,_ Sara Mehrabi and Lisbeth Jonsson *


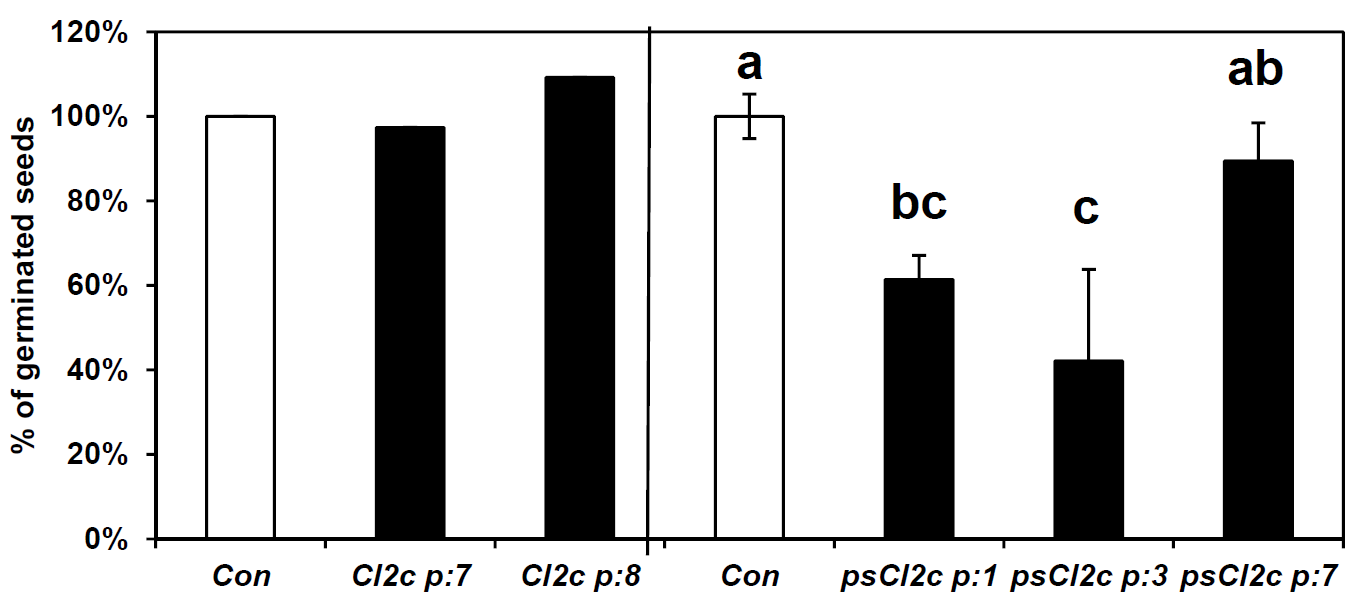


**Figure S1.** Germination percentage of azygous control and transgenic Arabidopsis seeds. Surface sterilized seeds were sown on ½ MS plates (30 seeds per plate, three plates per line). Results (± SE) represent the percentage of germinating seeds after 6 d normalized to control set as 100%. The average numbers of germinating seeds of control azygous plants for CI2c and psCI2c plants were 25 and 19, respectively. Bars carrying different letters are significantly different at *p ≤* 0.05 as determined by one-way ANOVA followed by Tukey HSD as a post hoc test.

| **(A)** | 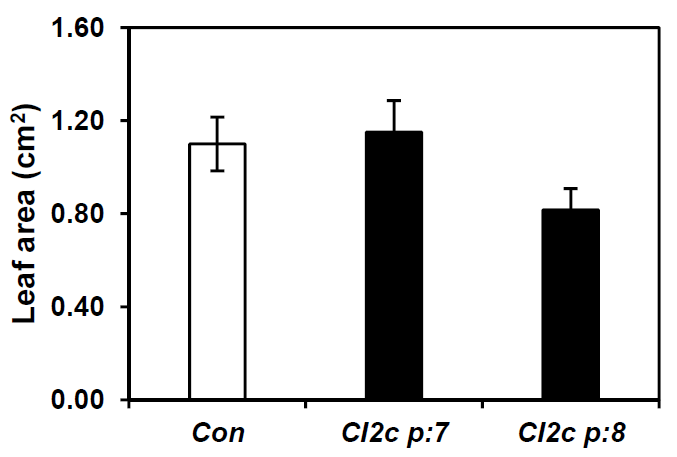 |
| --- | --- |
| **(B)** | 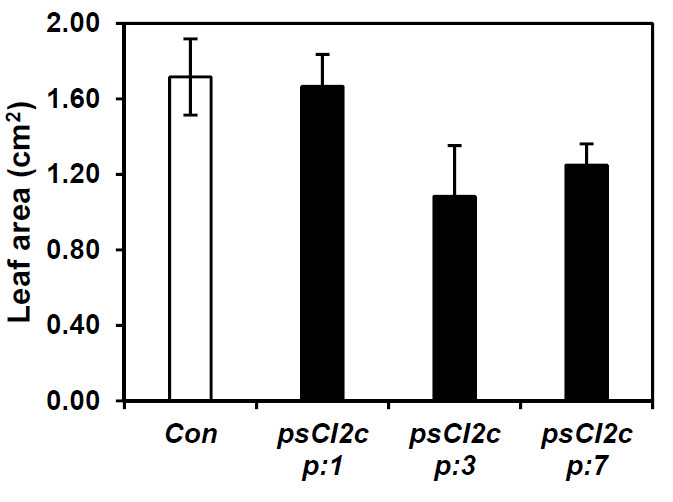 |

**Figure S2.** Leaf area of transgenic Arabidopsis as compared to azygous controls. Results (± SE) (*n* = 6) represent the average area of one of the youngest, fully developed leaves (leaves 5 or 6 from the top) of each transgenic and control line. Each measured leaf was from a different plant. (**A**) Control and *CI2c* transgenic plants, 24 d old; (**B**) control and *psCI2c* transgenic plants, 24 d old. Plants were grown in LD conditions. All measurements were done using ImageJ software (<https://imagej.net/>). There was no significant difference between the different lines (*p* > 0.05, one-way ANOVA).


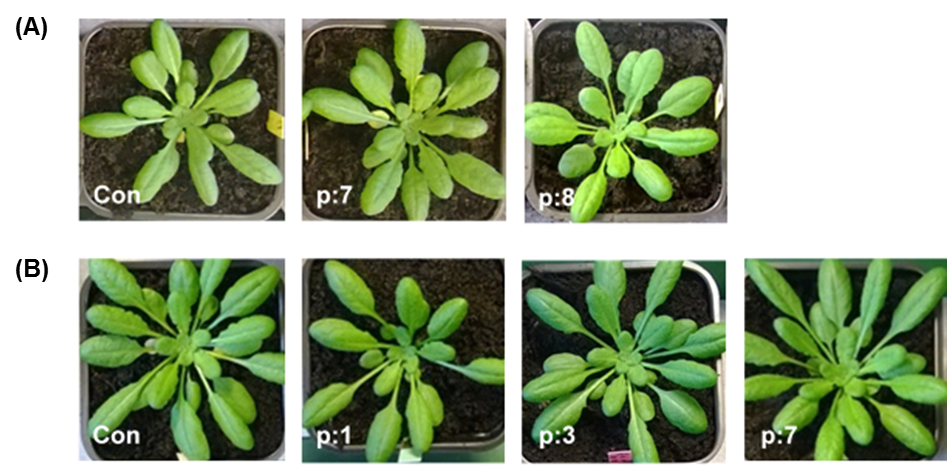


**Figure S3.** Phenotypes of transgenic plants as compared to azygous controls. (**A**) Control and *CI2c* transgenic plants, seven weeks old; (**B**) control and *psCI2c* transgenic plants, seven weeks old. Plants were grown in SD conditions.


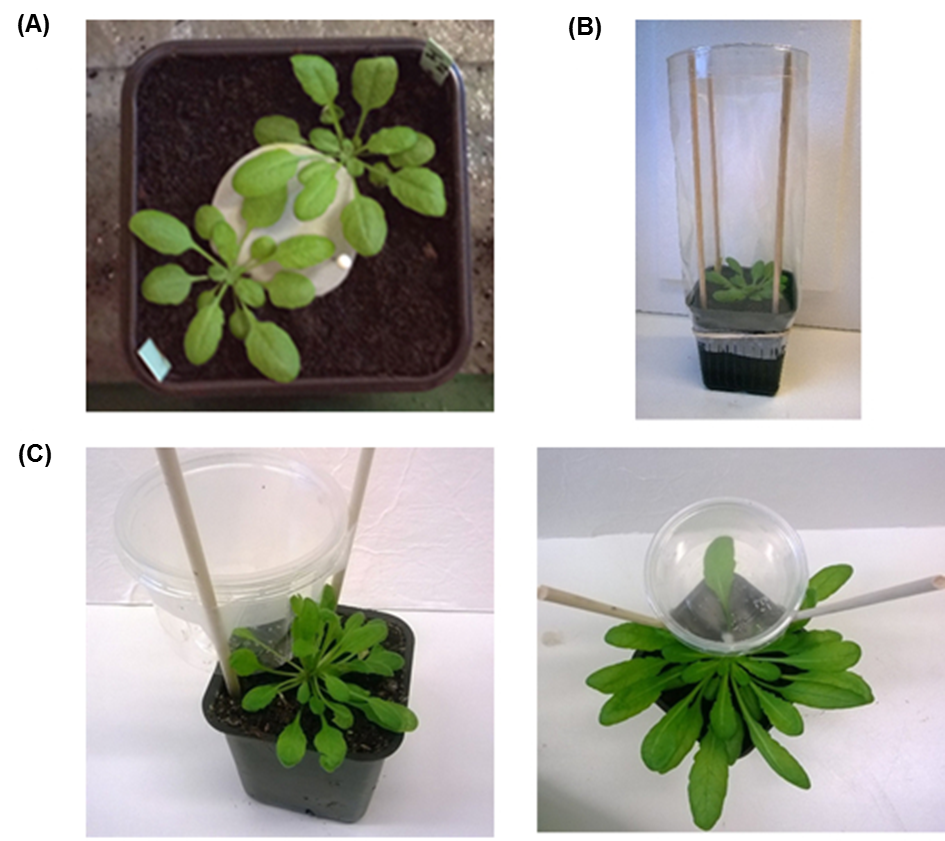


**Figure S4.** Aphid tests. (**A**) choice test. Azygous control and transgenic plants were planted in two corners of the same pot (7 × 7 cm). Twenty apterous *M. persicae* adults were released in the middle of the filter paper (3 cm diameter). Aphid location was assessed after 24 h; (**B**) fecundity test with nymphs (14 d test). Plants were enclosed under an aerated cage, composed of plastic and semi-transparent fabric; (**C**) life span test. One leaf from each plant was enclosed in a transparent plastic container (5 cm high, 7 cm diameter) with a small hole, so that the leaf could be placed inside. The cage was aerated by creating small holes in the plastic with a paper pin. The space around the petiole was closed with a piece of cotton wool to prevent aphid escape.

**Table S1.** **Flowering of transgenic plants as compared to control azygous lines**. Sterilized seeds were sown on ½ MS plates and replanted to soil after 14 days. Plants were grown in LD conditions. Results represent the percentage of plants that developed flowers and average (± SE) number of days before flowering; *n* = 10. The remaining plants did not develop flowers. There were no significant changes between different lines (*p >* 0.05, one-way ANOVA).

|  | **Con** | ***CI2c p:7*** | ***CI2c p:8*** | **Con** | ***psCI2c p:1*** | ***psCI2c p:3*** | ***psCI2c p:7*** |
| --- | --- | --- | --- | --- | --- | --- | --- |
| % Flowering plants | 90 | 90 | 90 | 90 | 100 | 80 | 100 |
| Days to flowering | 21 ± 0,29 | 21 ± 0,29 | 21 ± 0,34 | 21 ± 0,40 | 21 ± 0,37 | 20 ± 0,16 | 21 ± 0,31 |

**Table S2.** Primer sequences used in RT-qPCR.

| **Putative or Known Function** | **Gene Abbreviation** | **Accession No.** | **Primer Sequences** |
| --- | --- | --- | --- |
| Clathrin adaptor complexes medium subunit | *Clathrin* | NM_203166.2 | F AGCATCTGGTCTGCGAGTTC  R CACAATGGCTTAGAGATTCTGC |
| Proteinase inhibitor | *CI2c/BCI-7* | AJ250663.2 | F AGGTAGCGGGAAAGTCCATC  R GGTCCTGAAGTCGAGGGTCA |
| TIP41-like protein | *TIP41* | NM_119592 | F TCATGGTTCCTCCTCTTGCG  R ACGAAGAACAGTTGGTGCCT |
